# An odor detection system based on automatically trained mice by relative go no-go olfactory operant conditioning

**DOI:** 10.1038/srep10019

**Published:** 2015-05-06

**Authors:** Jing He, JingKuan Wei, Joshua D. Rizak, YanMei Chen, JianHong Wang, XinTian Hu, YuanYe Ma

**Affiliations:** 1 Key Laboratory of Animal Models and Human Disease Mechanisms, Kunming Institute of Zoology, Chinese Academy of Sciences, Kunming, Yunnan 650223, China; 2 University of the Chinese Academy of Sciences, Beijing 100049, China; 3 Medical Faculty, Kunming University of Science and Technology, Kunming, Yunnan 650550, China; 4 State Key Laboratory of Brain and Cognitive Science, Institute of Biophysics, Chinese Academy of Sciences, Beijing 100101, China; 5 Kunming Primate Research Center, Kunming Institute of Zoology, Chinese Academy of Sciences, Kunming, Yunnan 650223, China

## Abstract

Odor detection applications are needed by human societies in various circumstances. Rodent offers unique advantages in developing biologic odor detection systems. This report outlines a novel apparatus designed to train maximum 5 mice automatically to detect odors using a new olfactory, relative go no-go, operant conditioning paradigm. The new paradigm offers the chance to measure real-time reliability of individual animal’s detection behavior with changing responses. All of 15 water-deprivation mice were able to learn to respond to unpredictable delivering of the target odor with higher touch frequencies via a touch sensor. The mice were continually trained with decreasing concentrations of the target odor (*n*-butanol), the average correct percent significantly dropped when training at 0.01% solution concentration; the alarm algorithm showed excellent recognition of odor detection behavior of qualified mice group through training. Then, the alarm algorithm was repeatedly tested against simulated scenario for 4 blocks. The mice acted comparable to the training period during the tests, and provided total of 58 warnings for the target odor out of 59 random deliveries and 0 false alarm. The results suggest this odor detection method is promising for further development in respect to various types of odor detection applications.

Odor detection tasks in human society play an important role under a number of circumstances, such as in drug searches^1^, odor-based disease diagnosis^2^,^3^,^4^, and contraband searches^5^,^6^. A number of animals have been employed for these types of odor detection^1^,^7^,^8^,^9^,^10^. Dogs are the most commonly trained and publically known animals to perform these odor searches and detection tasks^7^,^11^. However, patrol dogs are not always appropriate for specific detection situations because they do not work without a human handler, may have a threatening effect, or have reduced olfactory efficiency while under overheating conditions^12^. Since animals as rodents^13^,^14^,^15^,^16^,^17^ and insects^18^,^19^,^20^ have good olfactory abilities, so these animals have also been trained in the odor detection tasks. For example, rats, within a laboratory setting, have been trained to search for contraband odors and have learned to rear on their hind legs when alerting to an odor of interest during their search^1^. Moreover, a commercialized mouse-based explosive detector created by Israeli start-up company BioExplorers has been reported with limited details^21^. In addition, insects, such as fixed moths or freely flying honeybees, have been trained to detect explosives and locating land mines^8^,^10^. However, although theoretically insects have olfactory ability to execute odor detection tasks^9^,^18^,^19^,^22^, but their tiny body size is both an advantage and a disadvantage, for their lifespan is shorter and weaker than rodents and need more refined reward control settings. Furthermore, animals in most of these indoor and outdoor detection tasks applications were trained manually with limited training efficiency.

Rodents can learn novel tasks and be trained to interact with a programmed machine, which does not require human handlers^23^,^24^. For example, rodents have been efficiently trained in olfactory operant conditioning tasks^25^,^26^, which have been applied to the investigations of olfaction related functions^13^,^15^. In these tasks, mice were trained to insert their nose into an odor sampling port to trigger odor delivering^23^,^27^,^28^,^29^, then to sample it quickly and adopt an action depending on different paradigms, e.g. the go no-go olfactory operant conditioning^23^,^28^, and the two-alternative forced-choice olfactory operant conditioning (2AC)^27^,^30^. These methods and experiments further convinced us that the mice can be ideal candidates for the development of odor detection systems, as their innate detection systems have extremely strong olfactory sensitivity and odor discriminatory ability^13^,^14^,^15^,^16^, their relative small sizes and high learning efficiency with automatic training device.

However, the existing training paradigms could not meet the challenges of application: First of all, in the natural detection situation an odor cue comes and goes at any time, but the odor in these experimental situations was initiated by the animal. Second, in a real-time monitoring condition with the changing signal-to-noise ratio of target odor and the fluctuation of animals’ states, how to control the detection behavioral reliability of the animals? In the situations with the pure binary responses (e.g. ‘go no-go’, ‘left or right’ (2AC), ‘escape or not escape’), the reliability could be guaranteed only if working animal numbers were enlarged (for example, in the BioExplorers detection system, they used “three concealed cartridges, each of which houses eight mice”) or the animals were severely trained to behave ideally, however since animal’s fluctuation of motivation, attention extent, etc., could hardly be eliminated, so the ideal performance of animal might be traded off with reduced training efficiency. So it proposed a dilemma between animal numbers and training efficiency in the condition of these paradigms. Next, the odor detection task needs multiple animals working together to function as a stable unit, and none of the published methods was set up for simultaneously training of multiple rodents. In another perspective, the existing operant conditioning paradigms were designed for research rather than application, so it reasonably results in the use of uniform training parameters in despite of individual difference, which might be at the expense of optimal training efficiency when aiming at application use.

Therefore, a new application-oriented system is needed to be designed to meet the challenges mentioned above, and look forward to meet the needs of odor detection in public applications ultimately. As such, the mouse-based odor detection system must detect target odor passively as the occasion demands; and the working animal numbers need to be minimized in the balance of the system’s effectiveness.

In this work, a novel apparatus and operant conditioning paradigm was designed as a training module for 5 mice to conduct odor detection such that the requirements of reliable odor detection can be better met, with the intention that its further application could be modified to meet the needs of odor detection, while reducing the limitations of current animal detection models.

## Materials and Methods

### Ethics statement

Fifteen adult male Kunming strain mice (90–100 days old at the beginning of the experiment) were purchased from the Chengdu Experimental Animal Center (Chengdu, Sichuan, China). Five mice were housed together under natural lighting conditions in a plastic cage, with temperature and humidity controlled to 25 °C and 40%. Food was available ad libitum.

Care and treatment of the mice were carried out in accordance with the guidelines for the National Care and Use of Animals approved by the National Animal Research Authority (P.R.China) and was approved by the Institutional Animal Care and Use Committee (IACUC) of Kunming Institute of Zoology.

### Water Restriction

**Figure 1:**
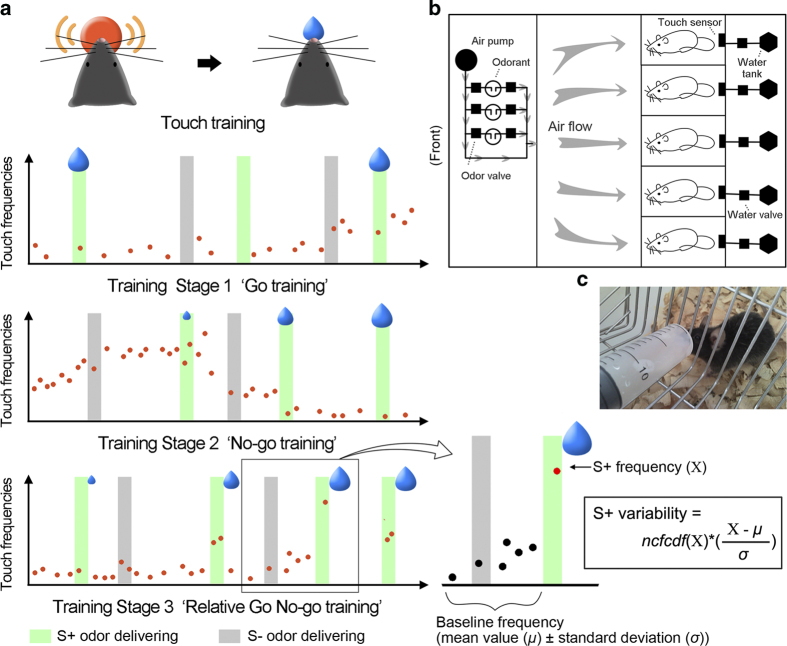
(**a**) Abridged general view of the training procedure. Orange dots indicate touch frequencies. Blue water-drops indicate conditional changing rewards. Details see the Method section. (**b**) A diagrammatic sketch of the odor detection device (top view). (**c**) Image of a mouse receiving water rationing before training. Figure drawn by J.H.

Training of the mice was performed via water restriction. One week prior to training, water deprivation was executed where each individual mouse had access to only 2.5 ml of water for 10 minutes per day in a separate cage (Fig. 1c). At the end of the 10 min, the remaining water was removed and the water consumption volume of each mouse was recorded. The average recorded value from last two days of deprivation was denoted as the daily requirement volume (DRV).

During the training period, the water reward level for each mouse was individually adjusted according to its DRV. On every training day, each mouse would receive systematic rewards according to its odor detection behaviors (described in detail below in the training procedure section). Following 3 training blocks of a day, the animal would receive complementary water up to 70% of its DRV. After every 4–6 days of training, the animals were given a 2-day rest period, where water was presented again for 10 min and the water consumption recorded as before. A new DRV was averaged from the rest days, which was used as a basis to adjust the reward level during training. These policies were designed to multilaterally control the motivation of the animals during training and to keep them healthy throughout the training.

### Odorants

The odorant used as the target odor was *n*-butanol (purity >99%, Sigma-Aldrich). The target odor was used as the positive stimulant (S+), and was presented with a reward. Mineral oil (M3516, Sigma-Aldrich) was used as both the solvent and as the negative stimulant (S−) control odor, which was delivered without a reward. 8 concentrations of the target odor were used (volume ratio): 100%, 10%, 3%, 1%, 0.3%, 0.1%, 0.03%, and 0.01%. The odorants were prepared fresh one day before usage and 2 mL of each dilution was used in each experiment. After each training day, the odorant was removed from the apparatus and replaced with new solutions on the next day.

### Apparatus

The training device was a cuboid shape constructed out of acrylic: 750 mm * 500 mm * 300 mm (length*width*height) (Fig. 1b). It was divided into four sections: 1) odor delivery control compartment; 2) animal training section containing five parallel closets to house 5 individual mice; 3) water control section; and 4) an electric circuit section for input/output signals.

The odor delivery control compartment contained 1 micro air pump (TYAP247, Ningbo Beilun Tiaoyue Machinery Co. Ltd., Zhejiang, China); 6 soundless solenoid valves (BLJSF101-24V, Cixi Kaida Electric Appliance Co. Ltd., Zhejiang, China; it uses a rubber valve plug, switches with little sound); and 3 glass sample bottles. In the section chamber, the air pump, which generated air flow (rate: 5 L/minute), was connected through a polyurethane tube to stainless steel T-shape connectors that branched into 4 parallel air pathways. Three of these paths were each controlled by two soundless solenoid valves and the other branch was always unimpeded. The solenoid valves were separately connected to input and output needles (20 G diameter), which pierced one of the three sample bottles, arranged in parallel. The output valve from each sample bottle was connected to a final customized glass outlet, which dispersed the respective odors to the second chamber for animal training. One of the air pathways (one sample bottle with mineral oil and two valves), was served as a shield one. It would switch randomly once every 5 ~ 35 seconds (except odor delivering periods) for 9 seconds’ duration to prevent artificial cueing of the mice to subtle sound generated by the switches of the odor controlling valves, and/or potential detection of mineral oil. The odor valves and the polyurethane tubes were all replaced with brand new ones when the odor concentration was changed.

The second chamber contained five mice closets with an individual size of 170 mm * 100 mm * 200 mm. The front wall of each closet were ventilated wire barriers; the separation walls of the closets were fully blocked from visual sight; and on the posterior wall there was a proximity switch (touch sensor, CH-18DO/DC 5(8)B, Zhongshan Lingchuang Sensor Factory, Guangdong, China) with an extra blue feedback LED, an exhaust fan (described below) and a water supply tube located 15 mm under the touch sensor.

Each of the 5 water supply tubes were connected to a water supply tank and were controlled by soundless solenoid valves located in the water control section of the apparatus.

The overall air flow throughout the whole device, external to the odorant delivery system, was maintained by three different types of fans located at different locations on the apparatus: 4 large wind volume fans (2806RL-04W-B59, Minebea-Matsushita Motor Corporation, Japan), located anterior to the odor delivery chamber; 2 smaller fans (2404KL-04W-B39, Minebea-Matsushita Motor Corporation, Japan), located posterior to the odor delivery chamber and adjacent to the odor delivery system outlet, to mix and spread the odor within the 5 animal training chambers; and 5 exhaust fans (BUB0512HB, Delta Electronics Public Co., Ltd, China) on the posterior wall of each mouse chamber to ensure continual air flow through the apparatus. During training, the apparatus was placed on the room’s ventilation system to exhaust the gas output quickly.

Furthermore, the fans made noise throughout the training, which drowned out any weak sound caused by the subtle movements of the water valves or odor valves, limiting these other sounds from being perceived or distinguished as alternative training cues by the mice.

The apparatus and training program was controlled by a protocol written in the Matlab software package (2009b). The parallel port of the PC was used for I/O signals and the electric circuit provided soundless operations.

### Training Procedure

A group of 5 mice were trained together simultaneously in the apparatus. There were 4 steps of training procedure (Fig. 1a). In the training stages, the mice were trained with a random presentations of S+/S− trials, where the animals received an inter-trial-interval (ITI) followed by a presentation of the mineral oil control (S− trial) or the target odor (S+ trial), and another ITI followed by another reiteration of the protocol. The settings of all training parameters (presentation times, proportion of S+ to S− presentations, etc.) in the different training stages are shown in Table 1.

### Touch training

Following the water restriction week, the mice were first trained to touch the sensor to get a drop of water (1/100 of an individual mouse’s daily requirement) for 2 training blocks. A training block was defined as up to 20 drops of water or 15 minutes within the training closet; whichever limit was reached first.

#### Training Stage 1: ‘Go training’

This step was aimed at establishing the connection between reward and the S+ stimulus by animals’ casual touches. A full reward (1/45 of individual mouse’s daily requirement) was delivered to the animal right after a mouse first touched the sensor during an S+ trial. The odor delivering time was gradually decreased trial by trial from 20 to 12 seconds for both S+ and S− trials.

For the first two training stages, a shared condition, termed ‘Keep-Away’ was used to refrain the mice from unceasingly touching the sensor. It restrained every trial from starting after each ITI until more than half number of mice in the group had refrained from touching the sensor for 5 seconds. The ‘5 seconds’ was determined appropriate from preliminary study as longer limits would result in long delays between odor presentations (up to tens of minutes) and shorter limits might have mislead mice to perform unceasing guess touches.

#### Training Stage 2: ‘No-go training’

In training stage 1, mice could get reward by relative high frequency guessing touches in despite of the S+ stimulus. Then, training stage 2 was designed to get rid of the ‘guessing touches’ which were the touches without the S+ stimulus delivering. Similar to stage 1, a reward was delivered right after the first sensor touch during an S+ trial. However, the reward volume of each S+ trial depended on two aspects, the past baseline frequency and the baseline frequency (described in detail below in Training Indices Measured): in other words, individual historical average ‘guessing touch’ frequency which was accumulated before a training block; and its average ‘guessing touch’ frequency right before a specific S+ trial. If the latter was lower than the former, which means less ‘guessing touches’ in comparison with itself, then it would result in a higher reward volume, and vice versa (calculation details see Table 1). The odor delivering time was decreased trial by trial from 12 to 10 seconds.

#### Training Stage 3: ‘Relative Go No-go training’

Mouse was trained to perform with significantly different touch frequencies between the S+ trials and other periods at this training stage. Different from stage 1 & 2, the reward was delayed and delivered at the end of an S+ trial. The reward volume was depended on an S+ variability value (described in detail below in Training Indices Measured, and also see Fig. 1a). Briefly, a full reward volume resulted when a relative high touch frequency during specific S+ trial occurred with a low touch frequency during a period before the S+ trial (calculation details see Table 1). The odor delivering time was 9 seconds for every trial.

### Training Indices Measured

The following training indices were measured for each individual mouse and used to evaluate odor detection of the mice in the experiments listed below:

#### Touch frequency and S+ frequency

The training program recorded every sensor touch and calculated the touch frequency repeatedly in 2 second intervals for the number of sensor touches occurring in a 10 second rolling time window (number of touches/10 s). A frequency-record was maintained for each 10 second period except for those generated during the S+ delivering period and 4 seconds after the S+ period. For an S+ trial, it was named the S+ frequency and refers to the number of sensor touches during a S+ odor delivering divided by the odor delivering time.

#### Baseline touch frequency (Baseline frequency)

The baseline frequency was a dynamic description of an individual mouse’s sensor touch pattern in real time in the absence of S+ odor presentation. The baseline frequency used for a single S+ delivering trial was confined to the most recent 30 frequency records (about 1 minute prior to the S+ trial). Every baseline frequency was calculated as a mean frequency value with a standard deviation. However in the text, ‘baseline frequency’ refers to its mean value if there was no other explanation.

#### S+ touch frequency variability (S+ variability)

firstly, the z-score value of an S+ frequency was calculated from the corresponding baseline frequency (restrict to the range of −3 to 3). Next, the z-score value was multiplied with cumulative possibility of the S+ frequency in its historical baseline frequencies’ non-central *F* distribution function (Matlab function *ncfcdf*, parameters were individually fitted by 200 trials’ baseline frequencies before a training block). As the results, the variability value would arise only if an S+ frequency was relative higher than both the current touch frequencies and the historical baseline frequencies. The graphic illustration of S+ variability was showed in Fig. 1a.

#### Correct response

A correct response was for an S+ trial if the S+ variability was equal or greater than 2. In addition, no judgments were carried out on S- trials because the touch frequencies of S- trials were included in baseline frequency determination and the data contributed to the correct responses computation.

#### Qualified/Failed mouse

Same ground as ‘correct response’ judgment, a mouse was determined to be ‘qualified’ of a task when the average S+ variability was equal or greater than 2; otherwise it would be a ‘failed mouse’ for certain task. It should be noticed that the S+ variability, correct response and qualified/failed mouse measurements are only meaningful for the training stage 3, namely the ‘relative go no-go training’.

#### The historical touch frequency of non-S+ period (Historical baseline frequency)

The historical baseline frequencies calculated as the mean value of the most recent 200-trial baseline frequencies for both the S- period and the ITIs combined. It was only used in the calculation of reward volume in training stage 2.

### Alarm Indices Measured

Different from the training indices, the alarm indices were calculated for monitoring and evaluating the reliability of qualified mice group’s touch behavior.

#### Touch variability

It was calculated using the same way as the S+ variability calculation, except the S+ frequency was replaced with the individual touch frequency in the most recent 10-second period.

#### AlarmIndex

It was calculated as the sum of 5 individual touch variability. A cumulative touch variability of 9 is compatible with a range of possibilities for 5 mice achieving high reliability of detection as a group, and the ROC analysis of the training data also showed AlarmIndex at 9 was the most sensitive and specific. Therefore, an AlarmIndex of 9 was set as the warning level threshold score for an alarm. In other words, the 5 mice needed to follow the target odor and synchronously touch the sensor with relative high frequencies to set off the alarm. A total potential AlarmIndex for all five mice was 15, where each animal had individual touch variability of 3.

### Experiments and Results

#### Experiment I. Training mice to follow the S+ odor presentation

To determine if the mice could learn to follow the S+ odor cue correctly in response to the training settings, 15 mice were trained to detect an odor in three stages, the data collected from the first 16 blocks of the training session, containing all stage 1, stage 2, and the first 6 blocks of stage 3 was processed and analyzed. The concentration of the target odor used in the S+ trials of stage 1, stage 2, and first 6 blocks of stage 3 was 100%.

All 15 mice learned to follow the target odor according to the average S+ variability in the last 3 blocks (mean S+ variability = 2.8636, standard deviation (SD) = 0.1495, n = 15).

**Figure 2:**
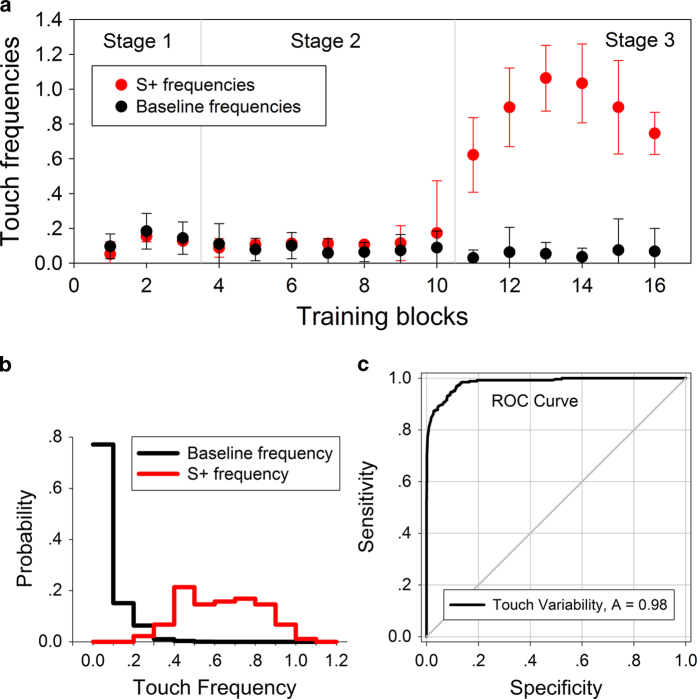
**Representative learning results of the mice.** (**a**) Learning curve of a mouse that qualified in training. (**b**) An example of the touch frequency distribution. (**c**) An example of ROC curve of an individual touch variability. Data in (**b**), (**c**) was from the same mouse training at 10% and 3% odor concentration blocks.

A typical learning process of a qualified mouse is shown in Fig. 2a. In the first two stages, water was delivered right after the first touch during every S+ odor presentation period. The touch frequencies in the S+ phase were at a low level. In the third stage, the reward was presented at the end of every S+ trial. The mice showed distinctly different touch frequencies between S+ trials and other periods (mean S+ frequency = 0.838, SD = 0.27; mean baseline frequency = 0.0692, SD = 0.0569, n = 15).

As the example showed in Fig. 2b, the possibility density distribution of the S+ frequency was obviously apart from the baseline frequency. Typically, the possibility density plot of a qualified mouse’s baseline frequency was distributed similar to the non-central F distribution.

#### Experiment II. Performance of mice training with decreasing concentrations of the target odor

All mice from experiment I were continually trained to detect to the target odor in descending concentrations (10%, 3%, 1%, 0.3%, 0.1%, 0.03% and 0.01%). Each concentration was trained in three blocks under the training parameters outlined in stage 3 (only last two blocks’ data of each concentration was used). An extra block was executed as mineral oil (S+ ) vs. mineral oil (S-) negative control block (0% concentration of the target odor). All fifteen mice were continually trained to the last block of experiment 2 no matter the mice could follow the odor or not.

**Figure 3:**
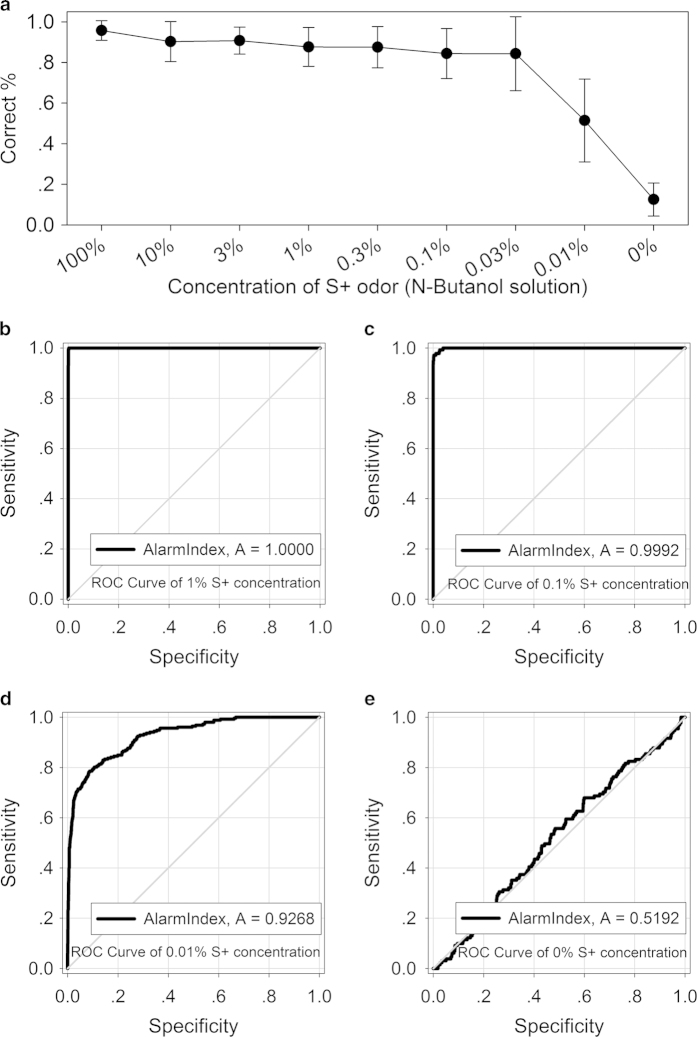
**Performance of the mice in detecting the target odor at gradually decreasing concentrations.** (**a**) Average correct percent of the mice, mean value ± SD. (**b**)–(**e**) ROC curve of mice groups detecting 1% (**b**), 0.1% (**c**), 0.01% (**d**), and 0% (**e**) concentration of the target odor. At each concentration, the animals were exposed to 3 repeating blocks (data only including last 2 blocks), except the 0% concentration was only exposed to 1 block.

Average correct percent (Fig. 3a) and S+ variability kept at the ideal level until training at 0.01% solution concentration (mean correct percent = 0.51, SD = 0.2; mean S+ variability = 1.75, SD = 0.56), and were around the chance level when training at 0% concentration (mean correct percent = 0.13, SD = 0.08; mean S+ variability = 0.13, SD = 0.31). 1 of the 15 mice occasionally did not cooperate during several training blocks; and the touch sensor would be dysfunction when it was wetted by mouse urine. So the influenced training block’s data was excluded from analysis. The sample size for calculation of average correct percent of decreasing concentrations is respectively: 15(100%), 14(10%), 14(3%), 13(1%), 15(0.3%), 12(0.1%), 15(0.03%), 15(0.01%), and 15(0%). Consequently, the group data would be valid only if it did not include exceptional individuals within a training block.

In parallel with training, the touch variability and the cumulative AlarmIndex for mice group were calculated and recorded every 2 seconds in real time; Receiver operating characteristic (ROC) analysis was applied to evaluate the sensitivity and the specificity of the AlarmIndex. As the concentration of S+ odor decreased, the ROC curve of AlarmIndex (all valid mice groups’ data of the same odor concentration was pooled together) changed from perfect to the chance level, as shown in Fig. 3b–e. ROC curve area respectively equals to 1.0000 (1%, sample size = 2856 time points), 0.9996 (0.3%, 4348), 0.9992 (0.1%, 2200), 0.9988 (0.03%, 4350), 0.9268 (0.01%, 4346), and 0.5192 (0%, 2204). Every S+ delivery typically including 3 sample points, S+ deliveries at the end of a block only including 1–2 sample points; first 4 seconds of S+ delivery and 10–14 seconds from S+ delivery, namely unstable rise and decline periods of AlarmIndex, were excluded from analysis.

#### Experiment III. The performance of the alarm algorithm in a simulated scene

Experiment 3 was performed to evaluate how the alarm algorithm would work with 5 qualified mice in a simulated detection scenario, when the target odor was sampled from outside of the device and water delivering was controlled by the mice’s voting results.

All mice from experiment 2 were trained again to detect target odor at a 0.3% concentration for 2 blocks. After retraining, a simulation detection task was conducted with any 5 qualified mice. In the test mode, one of two sample bottles (one with 0.3% *n*-butanol solution, the other one with mineral oil) was randomly chosen to be sampled at a 30–90 seconds’ interval. A polyurethane tube connected to the air pump was inserted into the selected bottle for 8 seconds per trial. No odor valve was used and the air within the bottle was synchronously presented to the 5 animals without impediment. The mice only received rewards if an AlarmIndex exceeded the warning level, and there was a 2.5 seconds’ reward delay after every first warning.

**Figure 4:**
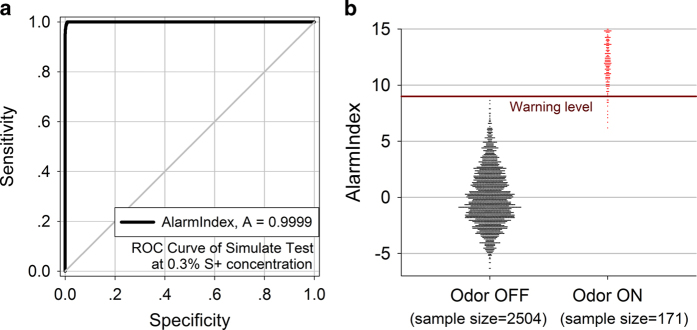
**ROC analysis of mice groups during simulate tests.** (**a**) ROC curve of simulate test at 0.3% target odor concentration. (**b**) Dot histogram of AlarmIndex across 4 blocks of the test.

Throughout four test blocks the target odor was delivered 59 times and the mineral oil was delivered 68 times over 5350 seconds test period (total 2675 sample time points). The performance of the mice in the odor detection test was generally similar to the performance of the mice in the training block at 0.3% concentration, Table 2. 58 alarms were correctly and quickly set off out of 59 presentations (AlarmIndex >=9). 1 presentation was missed; no false alarms were made by the mice. ROC analysis showed good odor detection ability of the mice group working with the alarm algorithm, Fig. 4 ROC Curve Area = 0.9999, Standard Error = 7.054e-5, 95% Confidence Interval 0.9997 to 1.000, *p* < 0.0001).

## Discussion

The current work provides a new solution of odor detection based on mice, which meet the challenges mentioned before. After 4–5 days’ simultaneously training of every 5 mice, all of 15 mice learned to follow the target odor cue; the mice could stably response to *n*-butanol at 0.03% solution concentration in the giving conditions; then with any 5 qualified mice working as a group, the alarm algorithm was tested in a simulated scenario. Combined results showed that the odor detection system could detect unpredictable target odor cue and set off an alarm within seconds.

The introduction of the novel measurements; S+ variability and touch variability, was the key of the odor detection system to provide stringent and reliable performance in odor detection. Fundamentally, S+/touch variability was calculated by comparing the touch frequency of individual mouse with both the current and the historical baseline frequency in real time, so the value could hardly be influenced by animal’s motivational increasing/decreasing touch frequencies. Therefore, S+/touch variability could honestly reflect the possibility of each individual detection event (Fig. 2c), and the possibility of detection would increase with higher touch variability. As a group, based on the individual touch variability, the possibility of detection increases with the number of mice showing high touch variability at the same time point.

Whether the mice were truly following the odor cue is inevitably being questioned. As described in the Materials and Methods section, there are multiple aspects to control animals’ behavior to follow the odor cue: the soundless solenoid valves, the shield odor valves, noise made by the fans, and the reward rules of training. The results of Experiment 1, 2, 3 demonstrated animals’ odor following reaction in different ways. Experiment 1 preliminarily proved that the qualified mice could follow the odor cue to take correct actions, but it could not completely exclude the potential that the qualified mice were following subtle different movements of the odor valves, although both of the S+ and the S- were controlled by same type of solenoid valve. Next, experiment 2 showed decreased detecting performance of the mice went along with decreased odor concentrations, this result could exclude the suspicion above. Furthermore, in experiment 3 the target odor was sample from outside of the device and no odor valve worked during the test, so the animals’ action might be led only by the odor cue. In addition, sample video showed that when the target odor was delivering, a qualified mouse always firstly turned towards the coming direction of the odor, then turned back to the touch sensor. This phenomena could hardly be explained by the cause of other sensory cues. As the possibility of interaction between the mice still existing: the visual sight between two neighbor mice was fully blocked; the 11 fans on the apparatus made much noise, so there was little chance for the mice to response correctly according to any sound cue made by group mates. Moreover, as showed in Fig. 2c, an individual touch variability achieved 0.98 ROC curve area, which means good individual detection ability of the target odor. All the existing evidences indicated that the qualified mice were actually following the target odor cue to act correctly.

As showed in Table 2, in the simulated test scenario the system was found to be as reliable as during the training period. The two false negative trials in the test (AlarmIndex = 8.71) and training (AlarmIndex = 8.31), might be result of motivation decreasing; 4 of the 5 false alarms during the training were happened at the mineral oil delivering periods. Although in the relative go no-go paradigm, touch responses following of S- odor might be punished by discount reward, since it was reported that mice could detect several kinds of mineral oil odor^31^; in one hand, the shield odor valves were designed to decrease the false alarm percent by increasing the frequent of mineral oil delivering; in the other hand, the alarm algorithm need to be more accurate to detect mice’s touch variability, and then the reliability of the system could be further improved.

Lower concentration of the target odor was not tested in a simulated test, because in the test mode water reward was controlled by the mice voting results; as the odor concentration decreased the possibility of unfair rewarding must be increasing to negatively influence the performance of the mice group. In real detection conditions, a target odor appears with a small chance, so the reward will not be controlled by the mice themselves in practice. In the training process, the AlarmIndex was only determined by the mice group’s touch behavior and it was independent with the control of odor delivering or water rewarding, so the sensitivity of the system could be better evaluated by the training results of decreasing odor concentrations, rather than a simulated test.

The lowest solution concentration of the target odor applied in experiment 2 was 0.01%, since the odor flow was spread into the inner space of the apparatus and delivered to 5 mice’s closets, so the actual concentration of the target odor detected by the mice was much lower than the headspace volatile concentration of the solution. However, the odor control part of the apparatus needs to be better refined in the future, such as the olfactometers used in previous studies^23^,^30^ so that the concentrations of odorants could be controlled in a more precise way.

The ability to use the innate olfaction system in mice in conjunction with mobile robotic apparatus will avail the sensitivity and reliability of this odor detection system to a wide-array of circumstances. Although multiple improvements and further tests are still needed, the relatively small working animal numbers and the efficient training paradigm will better allow mice to become readily available for odor detection under practical circumstances.

## Author Contributions

J.H., J.K.W. and J.R. wrote the main manuscript text, J.K.W. and J.H. did the experiments, J.H., J.K.W., J.W. and Y.C. analyzed the data and prepared the figures, J.H., Y.M. and X.H. conceived the experiments. All authors reviewed the manuscript.

## Additional Information

**How to cite this article**: He, J. *et al.* An odor detection system based on automatically trained mice by relative go no-go olfactory operant conditioning. *Sci. Rep.* **5**, 10019; doi: 10.1038/srep10019 (2015).

## Supplementary Material

Supplementary Video

## Figures and Tables

**Table 1:** **Training parameter settings for the different training stages**.

****	**Stage 1**			**Stage 2**							**Stage 3**
Containing blocks	**3 blocks**			**7 blocks**							**-**
Proportion of S+ trials	0.95	0.8	0.7	0.6	0.5	0.5	0.5	0.5	0.5	0.5	0.5
Number of trials per block	16	20	22	25	30	30	30	30	30	30	30
Odor delivering time(s) per trial	20→16	16→14	14→12	12→10	10	10	10	10	10	10	9
Reward mode	Immediately			Immediately							Delayed
Reward volume of each trial	F			F*(1-2*B-0.25*B/M + M)							F*V/3
ITI (s) (Randomly changed within the time range)	24 ~ 40			36 ~ 62							
‘Keep-Away’ Index (5 s)	Yes			Yes							No limit

F: Full reward volume (1/45 of individual mouse’s daily requirement); V: S+ variability; M: Historical baseline frequency; B: Baseline frequency. The dynamic reward volume was restricted by the range of 0 to full reward volume.

**Table 2:** **Odor detection and reporting behavior of groups of every 5 qualified mice as the odor detection test and training periods, respectively, both at 0.3% S+ odor concentration.**

	**Test**	**Training**
Warning delay (s)	5.86 ± 1.02	6.04 ± 1.16
First AlarmIndex	11.41 ± 1.44	11.23 ± 1.63
AlarmIndex peak value	12.81 ± 1.51	13.65 ± 1.38
Warning lasting time (s)	7.59 ± 2.03	8.96 ± 1.89
False alarm	0	5
Total time (s)	5350	8696
False negative	1	1
Total delivering of S+	59	82
